# Literal Pattern Analysis of Texts Written with the Multiple Form of Characters: A Comparative Study of the Human and Machine Styles

**DOI:** 10.3390/e28010036

**Published:** 2025-12-27

**Authors:** Kazuya Hayata

**Affiliations:** Sapporo Gakuin University, Ebetsu 069-8555, Japan; hayata@sgu.ac.jp

**Keywords:** literal pattern, normalized entropy, Simpson’s diversity index, chi-square test, backtranslation, machine translation, artificial intelligence

## Abstract

Aside from languages having no form of written expression, it is usually the case with every language on this planet that texts are written in a single character. But every rule has its exceptions. A very rare exception is Japanese, the texts of which are written in the three kinds of characters. In European languages, no one can find a text written in a mixture of the Latin, Cyrillic, and Greek alphabets. For several Japanese texts currently available, we conduct a quantitative analysis of how the three characters are mixed using a methodology based on a binary pattern approach to the sequence that has been generated by a procedure. Specifically, we consider two different texts in the former and present constitutions as well as a famous American story that has been translated at least 13 times into Japanese. For the latter, a comparison is made among the human translations and four machine translations by DeepL and Google Translate. As metrics of divergence and diversity, the Hellinger distance, chi-square value, normalized Shannon entropy, and Simpson’s diversity index are employed. Numerical results suggest that in terms of the entropy, the 17 translations consist of three clusters, and that overall, the machine-translated texts exhibit entropy higher than the human translations. The finding suggests that the present method can provide a tool useful for stylometry and author attribution. Finally, through comparison with the diversity index, capabilities of the entropic measure are confirmed. Lastly, in addition to the abovementioned texts, applicability to the Japanese version of the periodic table of elements is investigated.

## 1. Introduction

Japanese has a very peculiar writing system because the texts are written in three kinds of characters consisting of *kanji*, *hiragana*, and *katakana* [1]. Here, *kanji* is a Japanese name for Chinese characters. It is said that there are roughly 50,000 characters that were invented in ancient China, of which about 5000 (10%) have been used in the actual writing of Japanese texts. The characters were introduced into Japan around the first century. Dozens of them were in turn modified in a very cursive form, which results in *hiragana*, a system of syllabary consisting of about 50 characters. At the same time, Chinese characters were simplified substantially by taking a radical of the original character, which produced *katakana*, another syllabary system that is used along with *hiragana*. The writing in two kinds of *kana*’s mixed with Chinese characters, i.e., the composition written in *kanji* and *kana*’s, has been preserved since the Nara period (710–794). Although there is no strict rule of how to blend the three kinds of characters, writers are bound by a few implicit rules. For example, (1) Loan words from China are in principle written in *kanji*; (2) As for an auxiliary verb, a postpositional word functioning as an auxiliary to a main word, and the ending of a declinable word, *hiragana* should be used; and (3) Foreign loanwords except for those of Chinese origin as well as both onomatopoeia and mimesis should be transliterated into *katakana*. It should be stressed here that this peculiar writing system has been established spontaneously rather than as a language policy for the language. This history shows a striking contrast to the situation in other Asian countries where *kanji* had been used. For instance, Korean and Vietnamese declined the *kanji* usage entirely, where *kanji* was replaced by the Hangul alphabet and the modified Latin script, respectively. Xi Xia (1038–1227), an ancient country around China, had invented a strange system consisting of more than 6000 Chinese-like characters (pseudo-*kanji*), instead of using conventional characters of Chinese origin. Incidentally, the principal reason of preserving the multiple form of characters in Japanese texts can be inferred from the fact that in contrast to most of the world languages, Japanese texts do not leave a space between words, which results in difficulty to read texts written entirely in *kana* without any *kanji*.

In this paper, in the context of exploring novel applications of the entropy-based time series analysis, for several Japanese texts we conduct a quantitative analysis of how the three characters consisting of *kanji*, *hiragana*, and *katakana* are blended using the binary-patterns-based approach to the 6-bit sequence. In contrast to many successful applications to natural and social sciences [2,3,4,5,6], attempts to investigate the capability of the methodology for solving problems in the humanities are very limited [7,8]. Specifically, we take two different texts in the former and present constitutions of Japan as well as a short story written by Edgar Allan Poe (1809–1849) [9], which has been translated at least thirteen times into Japanese since 1929 [10,11,12,13,14,15,16,17,18,19,20,21,22]. For the latter, a comparison is made among all human translations currently available and the four machine translations by DeepL and Google Translate. As metrics of divergence and diversity, the Hellinger distance, chi-square statistic, normalized entropy, and Simpson’s index are employed. In particular, numerical results of the entropy reveal a few interesting features. Specifically, there are three clusters that emerge in the 17 translations, and the machine-translated texts exhibit entropy higher than the human translations. In the context of stylometry and author attribution, it will be interesting to suggest that our method can provide a tool useful for detecting whether a certain text was written by a human or generated by artificial intelligence. Finally, through a comparison between the two diversity indices, capabilities of the entropic measure are investigated. In addition to the abovementioned texts, numerical results of the Japanese version of the periodic table of elements are also given.

## 2. Method

**Figure 1:**
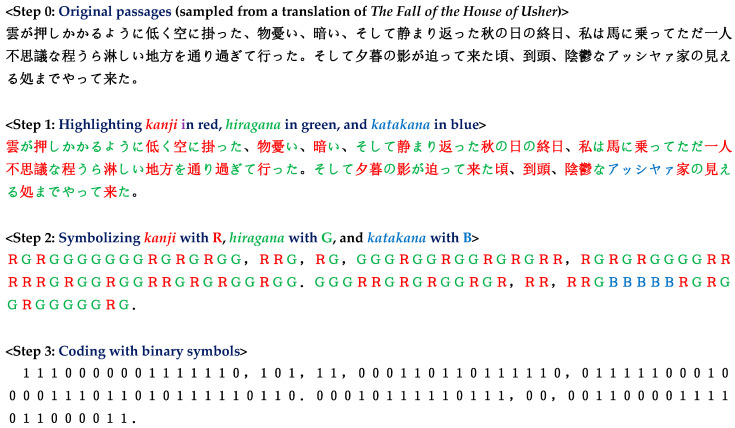
Procedure of obtaining binary sequence. There are three kinds of characters: *kanji* (Chinese characters), *hiragana*, and *katakana*, which are highlighted in red, green, and blue, respectively, in Step 1. The binary sequence in the final step is generated according to the rule of Equation (1).

**Figure 2:**
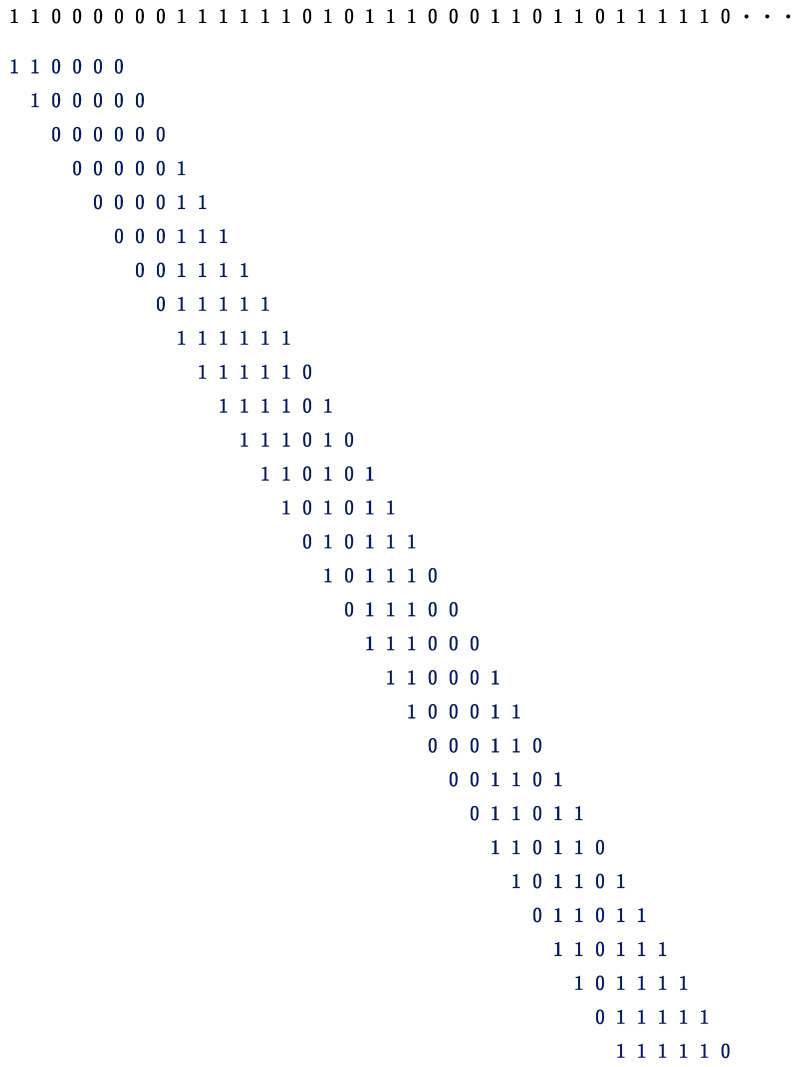
An example for explaining how to generate 6-bit binary codes from the opening data in Step 3 of Figure 1. Punctuation marks in Figure 1 are dropped.

The process necessary for generating a binary sequence from a Japanese writing (Step 0) is illustrated in Figure 1. First, the three forms of characters in the original passage are marked with different colors (Step 1). Subsequently, the three kinds of characters are symbolized with R, G, and B (Step 2). Next, the binary sequence *s*_1_*s*_2_ … *s_n_* is produced according to the rule (Step 3)

(1) $$s_{i}=\left\{\;\begin{array}{c} 0\;\;\;\;\;\;\;\;\;\;\;\;\;\;\;\;for\;x_{i+1}=x_{i}\\ \;1\;\;\;\;\;\;\;\;\;\;\;\;\;\;\;\;for\;x_{i+1}\neq x_{i} \end{array}\right.\;\;\;\;\;\;\;\;\;\;\;$$

for *i* = 1, 2, …, *n*, where *x_i_* (*i* = 1, 2, …, *n* + 1) represents R, G, and B in Step 2, and *n* indicates the length of the binary sequence. Finally, one can obtain *n* − 5 binary codes from the sequence of Step 3. An example for explaining how to generate 6-bit binary sequences from the head part (35 bit from the start) in Step 3 is shown in Figure 2. As metrics of divergence, we will employ the Hellinger distance defined by

(2) $$D_{H\;}=\sum_{i=1}^{m}(\sqrt{p_{i}}-\sqrt{q_{i}\;}{)}^{2},\;$$

where *p_i_* and *q_i_* (*i* = 1 to *m*) represent the relative frequencies of *C_i_* (*i* = 1, 2, …, *m*) in the two sequences to be compared, *m* denotes the number of categories of the code (for 6-bit coding, *m* = 2^6^ = 64), and the unit of the distance is nat, which abbreviates natural unit. Specifically, for this coding system we adopt the usual lexicographic order, i.e., *C*_1_ = 000000, *C*_2_ = 000001, … …, *C*_64_ = 111111.

In order to implement a nonparametric test, the chi-square value will be useful:

(3) $$\chi^{2}=\;\sum_{i=1}^{m}\frac{(f_{i}-F_{i}{)}^{2}}{F_{i}}.\;\;$$

Here, *f*_i_ and *F*_i_ (*i* = 1 to *m*) represent the surveyed and expected frequencies, respectively. Note that to avoid singularity, *F_i_* ≠ 0 unless *f_i_* vanishes. The relations between the relative frequencies in Equation (2) and the frequencies in Equation (3) are *p_i_* = *f_i_*/(*n* − 5) and *q_i_* = *F_i_*/(*n −* 5). With a theory of the combinatorial probability the expected frequencies can be calculated with the formulae

(4a) F1=n−5DM6,

(4b) Fi=n−56DM5N−M1      for i=2, 3, 5, 9, 17, 33,

(4c) Fi=n−515DM4N−M2     for i=4, 6, 7, 10, 11, 13, 18, 19, 21, 25, 34, 35, 37, 41, 49,

(4d) Fi=n−520DM3N−M3        for i=8, 12, 14, 15, 20, 22, 23, 26, 27, 29, 36, 38, 39, 42, 43, 45, 50, 51, 53, 57,

(4e) Fi=n−515DM2N−M4     for i=16, 24, 28, 30, 31, 40, 44, 46, 47, 52, 54, 55, 58, 59, 61,

(4f) Fi=n−56DM1N−M5     for i=32, 48, 56, 60, 62, 63,

(4g) F64=n−5DN−M6,

(4h) D=N6.

Here *M* and *N*, respectively, are the total of ‘0’ and the grand total of ‘0’ and ‘1’. Note that for the 6-bit coding *N* = 6 (*n* − 5).

Finally, to quantify the diversity of the code spectra the normalized entropy *h* (0 ≤ *h* ≤ 1), which is defined with the Shannon entropy, will be adopted

(5) $$h=-\frac{\sum_{i=1}^{64}p_{i}log{\;p}_{i}}{6\;log2},$$

where the 6-bit binary coding is implied.

## 3. Results

### 3.1. The Preamble to the Constitution of Japan

**Figure 3:**
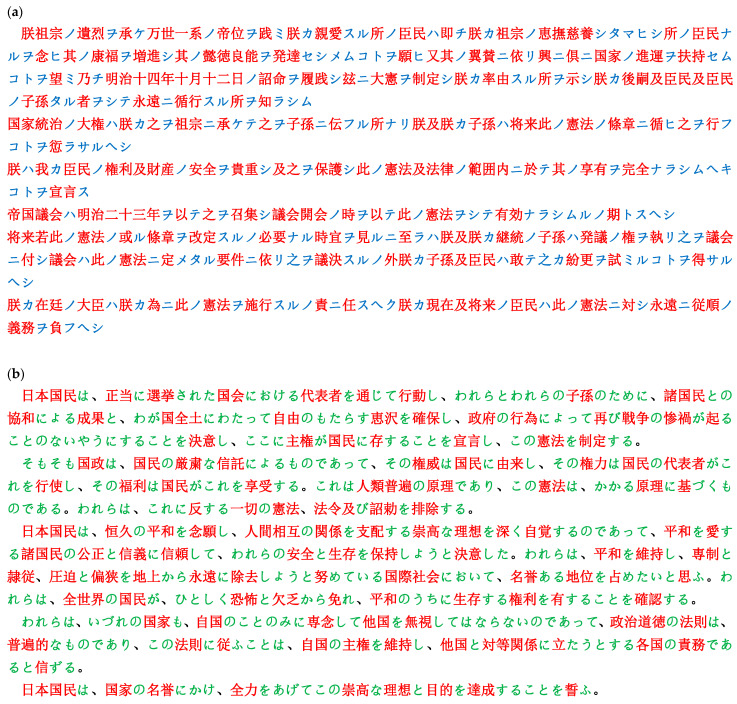
The Preamble to the Constitution of Japan. (**a**) The former constitution (promulgated on 11 February 1889). (**b**) The present constitution (promulgated on 3 November 1946). Note that each script displays a golden mean between arrangements of *kanji* and *kana*.

First, we consider two kinds of texts that correspond to the Preamble to the Constitution of Japan. One is a passage equivalent to a preamble to the Constitution of the Great Empire of Japan (the so-called Meiji Constitution), which was promulgated on 11 February 1889 and came into effect on 29 November 1890; the other is the Preamble to the Constitution of Japan, which was promulgated on 3 November 1946 and came into effect on 3 May 1947. These are given in Figure 3a,b, respectively. Here, the former is written in *kanji* and *katakana*, while the latter in *kanji* and *hiragana*. In this section we will investigate the ability of machine translations currently available, through comparison among original texts and those back-translated via English version into Japanese. The procedure of the backtranslation is explained as follows: (1) First, using a machine-translation device the original Japanese text is translated into English; and (2) The text just translated into English is subsequently translated into Japanese by the same device, which results in a Japanese text different from the original one. It should be mentioned here that, describing strictly, the abovementioned backtranslation will be made solely for the former text. For the present text, in addition to the Japanese text there has been an English text being available, the latter of which was provided by the Far Eastern Commission of the GHQ in the chaos directly after the end of the Second World War. For this reason, as for the present constitution, instead of the first step above, we will use the *original* English text in the translation into Japanese.

**Figure 4:**
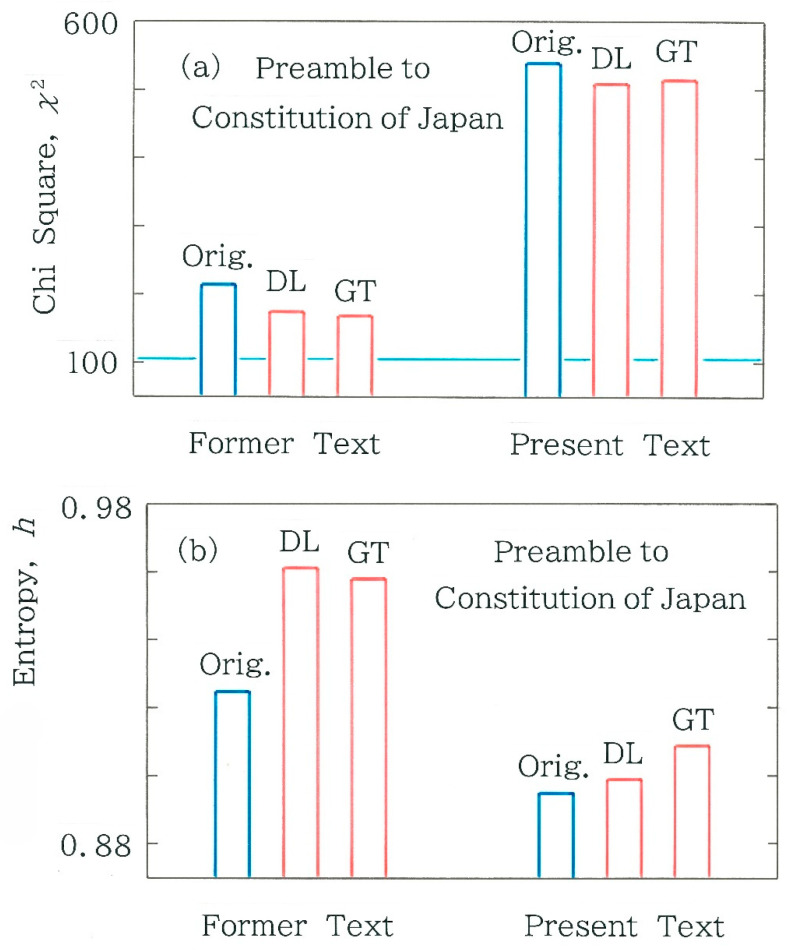
Comparison of metrics between the backtranslation and the Japanese original of the Preamble to the Constitution of Japan. Red bars indicate the backtranslations using DeepL (DL) and Google Translate (GT) while blue bars the Japanese original. The length *n* of the binary sequence is, in order from left to right, 510, 494, and 490 for the former text, while 599, 592, and 596 for the present text. (**a**) Chi-square statistic. The horizontal line in light blue indicates the critical value for the significance level *α*= 0.001. (**b**) Normalized Shannon entropy.

For the chi-square statistic and the normalized entropy, respectively, computed results are shown in Figure 4a,b. Here, the blue bars indicate the results of the original text, while the red bars indicate those of the backtranslations by DeepL (DL) and Google Translate (GT). First of all, we notice three features in Figure 4a: (1) In all six cases the null hypothesis is rejected (with significance level α = 0.001), demonstrating that the mixture of the two symbols (‘0’ and ‘1’) in the binary sequence is far from stochastic; (2) The chi-square values of the present text are substantially larger than those of the former text; and (3) For both former and present texts the chi-square value of the original is larger than that of the back-translated text. On the other hand, one can find two features in Figure 4b: (1) The normalized entropy of the present text is lower than that of the former one; and (2) For both former and present texts the entropy of the original is lower than that of the back-translated text. The principal reason will be attributable to a psychological bias of a lawmaker who tends to avoid a random arrangement of *kanji*.

**Figure 5:**
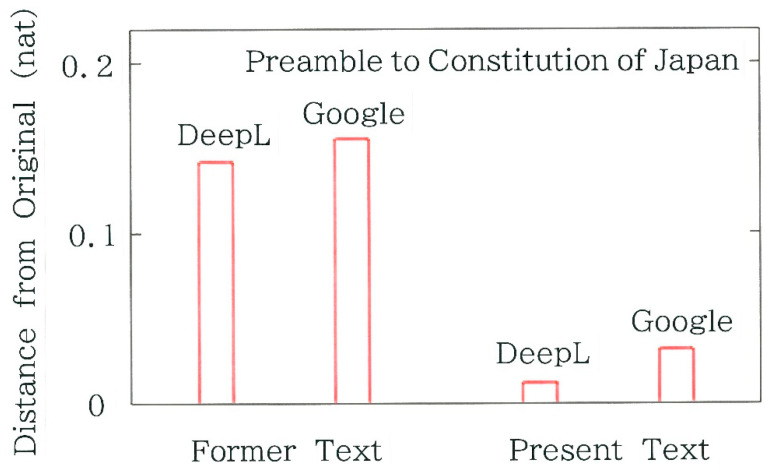
Hellinger distance from the original of the Preamble to the Constitution of Japan.

Computed results of the Hellinger distance from the original are shown in Figure 5. It can be seen that for both machine-translation devices the distance for the present text becomes much shorter than that of the former text. In comparison between the two devices, the distance for DeepL is slightly shorter than the counterpart for Google Translate, suggesting that the former possesses an ability slightly better than the latter. The significance of these results will be tested in Section 4.1.

### 3.2. Passages from an English Story

**Figure 6:**
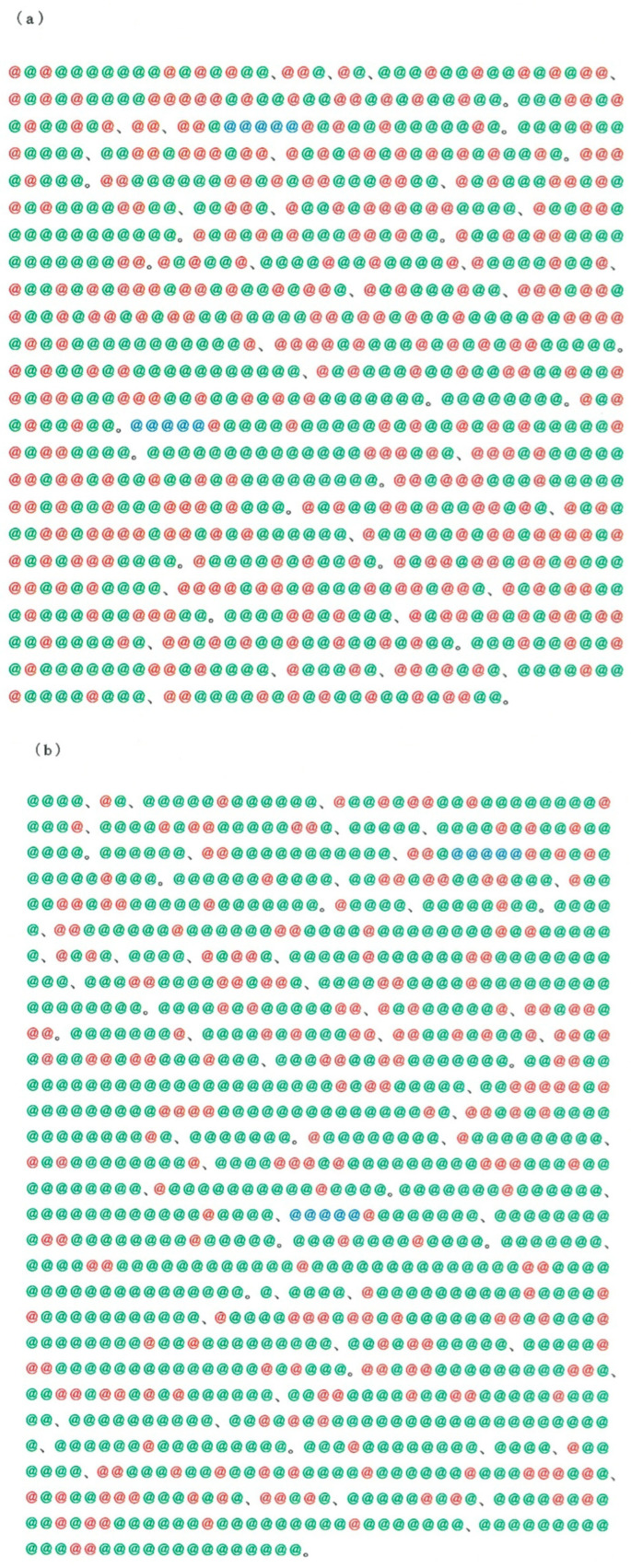
A bird’s-eye view of two Japanese texts, where red, green, and blue marks represent *kanji* (Chinese characters), *hiragana*, and *katakana*, respectively. (**a**) Writing by Tanizaki in 1929 [10]. (**b**) Writing by Koizumi in 1976 [17].

Instead of the above backtranslation into Japanese, in this section we consider the usual translation of an English text into Japanese. Here we concentrate on the opening paragraph in *The Fall of the House of Usher* [9], a short story written by Edgar Allan Poe (1809–1849). This story written in English has been translated at least 13 times into Japanese over the past 92 years [10,11,12,13,14,15,16,17,18,19,20,21,22], but the mixing between the three kinds of characters is substantially dependent on the style of each individual translator. In order to make an appeal to the eyes, in Figure 6, bird’s-eye views of the writing are compared for the translations by Tanizaki in 1929 [10] (*h* = 0.9688) and by Koizumi in 1976 [17] (*h* = 0.7997), which exhibit the maximum and minimum entropy, respectively. As was mentioned in Section 1, there is no strict rule concerning how to blend the three kinds of characters being highlighted in red, green, and blue, but writers are bound by a few implicit rules. That is, loan words from China are in principle written in *kanji* (marked in red); as for an auxiliary verb, a postpositional word functioning as an auxiliary to a main word, and the ending of a declinable word, *hiragana* (marked in green) should be used; and foreign loanwords except the Chinese origin should be transliterated into *katakana* (marked in blue). It appears that Tanizaki’s writing (as of 1929) in Figure 6a is out of style, while Koizumi’s writing (as of 1976) is occupied with too many *hiragana* to read fluently. Therefore, it can be conjectured that there is a compromise between the two extremes. The motivation of our study is to find the golden mean through calculation of the statistical metrics. Again, the two plots in Figure 6a, b show a marked difference in the mixing of the three kinds of characters. That is, the former contains a number of *kanji* (highlighted in red), whereas the latter contains a great deal of *hiragana* (highlighted in green). Note that other 11 translations [11,12,13,14,15,16,18,19,20,21,22] are intermediate between the two extremes. Incidentally, along with the comparative views in Figure 3a, b, those seen in Figure 6 are consistent with the statistical finding that the rate of *kanji* usage declined until the mid-20th century [23].

**Figure 7:**
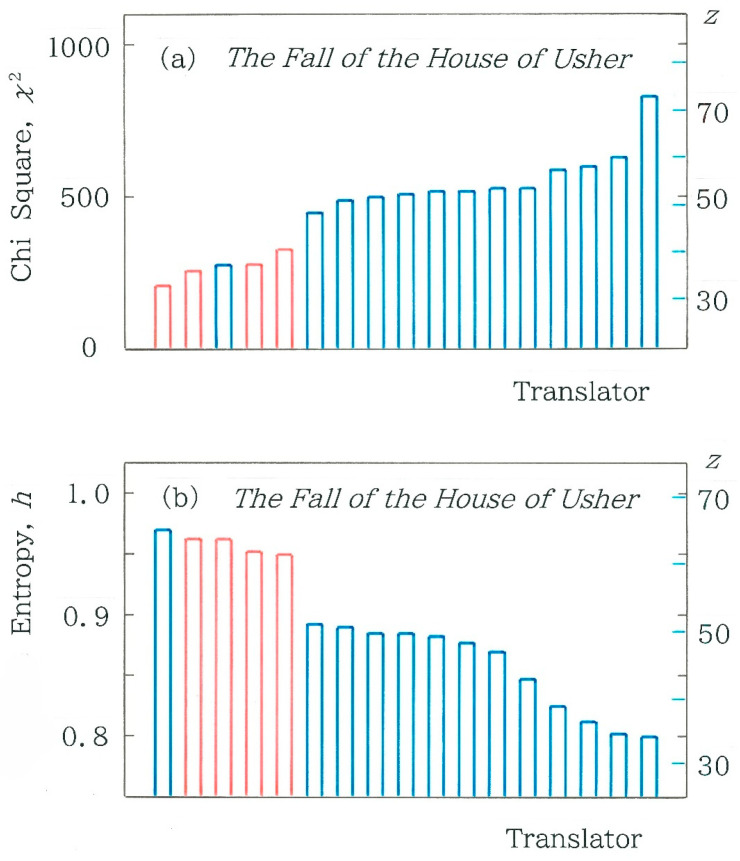
Comparison among metrics for 17 Japanese translations of the opening paragraph in *The Fall of the House of Usher* written by Edgar Allan Poe. The blue and red bars indicate the human and machine translations, respectively. The numerals on the right axis of ordinates indicate the standard scores (Equation (6)). (**a**) Chi-square statistic. The length of the sequence, *n*, is, in order from the left to right, 751, 872, 905, 817, 789, 854, 1040, 909, 918, 1080, 1006, 940, 923, 1103, 997, 1113, and 1212. (**b**) Normalized entropy.

In Figure 7a, we compare the chi-square values for 17 Japanese translations of the opening paragraph. The blue and red bars indicate the human and machine translations, respectively. First of all, it should be mentioned that in all 17 cases, null hypothesis is rejected (α = 0.001). As for the machine translations (red bars) in Figure 7a, in order from left to right, we can see DeepL (as of July 2025), Google Translate 1 (GT1; as of December 2023), GT2 (as of May 2025), and GT3 (as of July 2025). As for the human translations (blue bars), of the 13 translations, there exists a single exception (corresponding to Tanizaki’s translation [10]) being sited between GT1 and GT2. The tallest bar on the right extreme corresponds to the translation by Matsumura [11]. The scale on the right axis of ordinates indicates the standard score

(6) $$z=\frac{10\;(y-\bar{y)}}{s}+50,$$

where *y* represents the variable on the left axis of ordinates; $\bar{y}$ and *s*, respectively, are the mean and the standard deviation of *y*.

In Figure 7b, a comparison is made among the values of the normalized entropy for the 17 Japanese translations of the opening paragraph. There are five features being observed in this plot: (1) The translations can be divided into three clusters; (2) Across the first (leftmost) and second (intermediate) clusters, a steep discontinuity (Δ*h* = 0.0581) can be seen; (3) All of the 4 machine translations in red belong to the first cluster that exhibits relatively high entropy (0.950 < *h* < 0.962); (4) Of the 17 bars, the translation by Tanizaki [10] (the leftmost blue bar) preserves the highest entropy (*h* = 0.9688); and (5) The range of *h* is 0.1711.

Finally, we should mention the reason why the machine translations preserve relatively high entropy. Without exception, writings to be published by a publisher are subjected to an inspection by editors, who want to raise the popularity of the text as much as possible, and consequently will tend to avoid an excessive usage of *kanji* along with their psychological bias to avoid a too irregular arrangement of words written with *kanji*. Here, it should be noted again that the rate of *kanji*’s usage declined until the mid-20th century [23]. The reduction in the contents of *kanji* will give rise to the lower entropy due to the enhanced frequency of the first code *C*_1_. In striking contrast to the commercial effort for the human translations being published, the machine translations in Figure 7 (highlighted in red) are not subjected to a sort of inspection at all, because in radical contrast to humans, machines are free from any psychological bias. Note that this explanation is compatible with the observation in Figure 4b.

**Figure 8:**
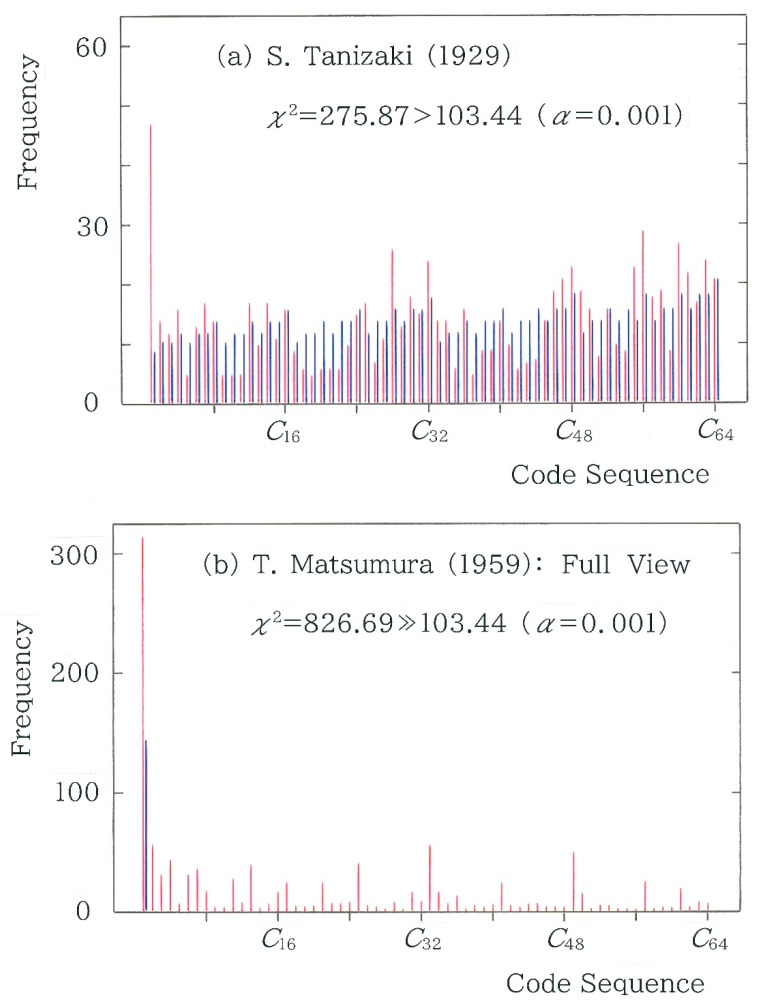

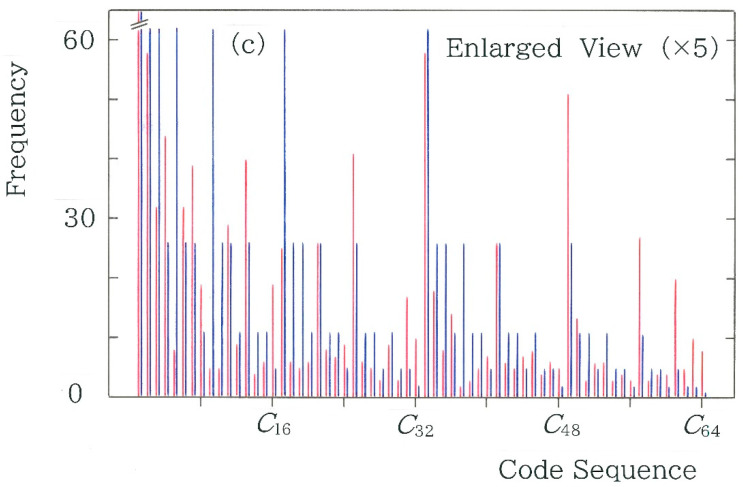
Code spectrum of the 6-bit binary sequence. The crimson and navy bars indicate the surveyed and expected frequencies, respectively. (**a**) Translation by Seiji Tanizaki [10]. (**b**) Translation by Tatsuo Matsumura [11]: Full view. Note that the expected frequency is juxtaposed in navy solely for the first code *C*_1_ = 000000. (**c**) Translation by Tatsuo Matsumura [11]: Enlarged view (×5). Note that the heads of the twin-colored bars for *C*_1_ at the left extreme are truncated.

To reveal the difference between the surveyed and expected frequencies, in Figure 8, frequency distributions of the 6-bit binary codes *C_i_* (*i*= 1 to 64) are shown in crimson (surveyed) and navy (expected), where two human translators have been chosen from the blue bars at the left and right extremes in Figure 7a, respectively: Seiji Tanizaki [10] (see Figure 8a) and Tatsuo Matsumura [11] (see Figure 8b,c). First, it can be seen that irrespective of the translators, there is a substantial difference between the heights of the twin dichromic bars. Secondly, in contrast with a relatively gentle rise and fall in the expected spectra (in navy), the envelope of the surveyed ones (in crimson) displays full of ups and downs as though stock prices were fluctuating violently. Thirdly, in comparison between Figure 8a–c, overall, the difference between the surveyed and expected frequencies is larger in the latter, which is consistent with the substantial difference in the chi-square statistic, i.e., $\chi^{2}\;$ = 275.87 for Tanizaki (Figure 8a), while $\chi^{2}\;$ = 826.69 for Matsumura (Figure 8b,c). To this subsection there is a postscript added in Appendix A.

## 4. Discussion

### 4.1. Tests of Statistical Significance

**Figure 9:**
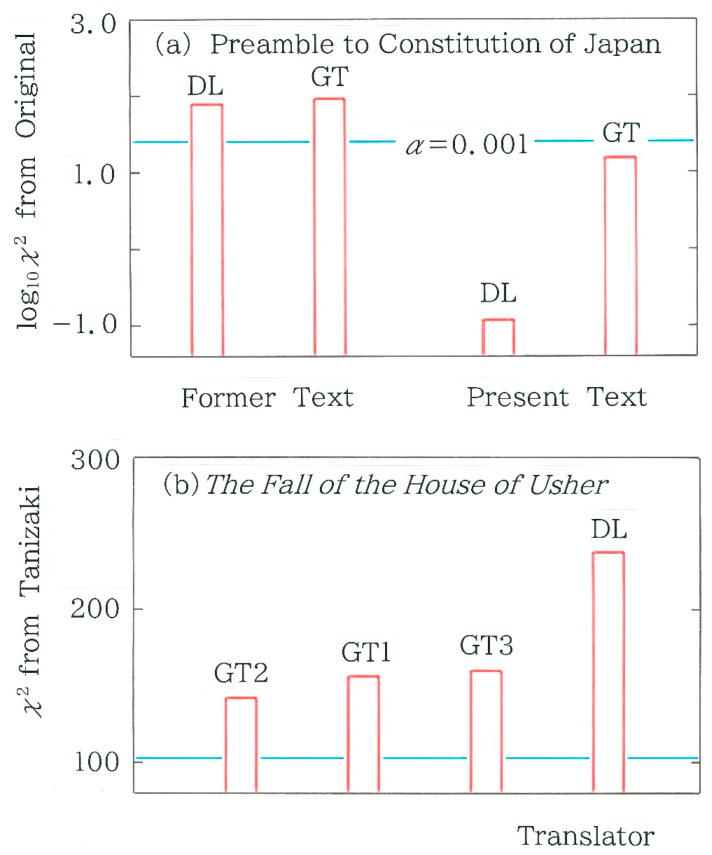
Comparison of chi-square values between texts translated into Japanese by DeepL (DL) and Google Translate (GT). The horizontal line in light blue indicates the critical value for the significance level *α* = 0.001. (**a**) Translations from the English version of the Preamble to the Constitution of Japan. Note that instead of the 6-bit coding adopted in Figure 5, the 3-bit coding is used. (**b**) Japanese translations [10,11,12,13,14,15,16,17,18,19,20,21,22] of the opening paragraph in *The Fall of the House of Usher* written by Edgar Allan Poe [9]. As in Figure 7, the 6-bit coding is preserved.

The results in Figure 5 concerning the Preamble to the Constitution of Japan have shown that irrespective of the device for machine translation, for the present text the Hellinger distance from the original becomes much shorter than that for the former text. To inspect whether the distance from the original text is statistically significant, we will conduct a test, where the expected frequencies of Equation (4) are replaced by the surveyed ones for the original text. Here, instead of the 6-bit binary coding (*m* = 64), we will adopt the 3-bit coding (*m* = 8) because for the former coding the necessary condition for the testing (i.e., all frequencies of expectation must not lower than 5) cannot be met. The results of the testing are shown in Figure 9a, which exhibit a striking contrast between the former and present texts. That is, for the former the null hypothesis is rejected with the significance level α= 0.001, whereas for the latter it cannot be rejected. In particular, it is surprising that the chi-square value for DeepL becomes as small as 0.118, which is found to be extremely smaller than the value 15.430 for Google Translate.

Next, we will revisit the results shown in Figure 7a, where the blue bar of Tanizaki was seen in the middle of the four red bars of machine translations. From these results, it appears interesting to investigate whether the difference between Tanizaki’s translation [10] and each machine translation is statistically significant. Computed results for the chi-square values from Tanizaki’s translation are shown in Figure 9b where the expected frequencies of Equation (4) are replaced with the surveyed ones for Tanizaki’s text. Evidently, in all four cases in the figure the null hypothesis is rejected (α= 0.001).

### 4.2. Scattergrams for Hellinger Distance and Entropy

**Figure 10:**
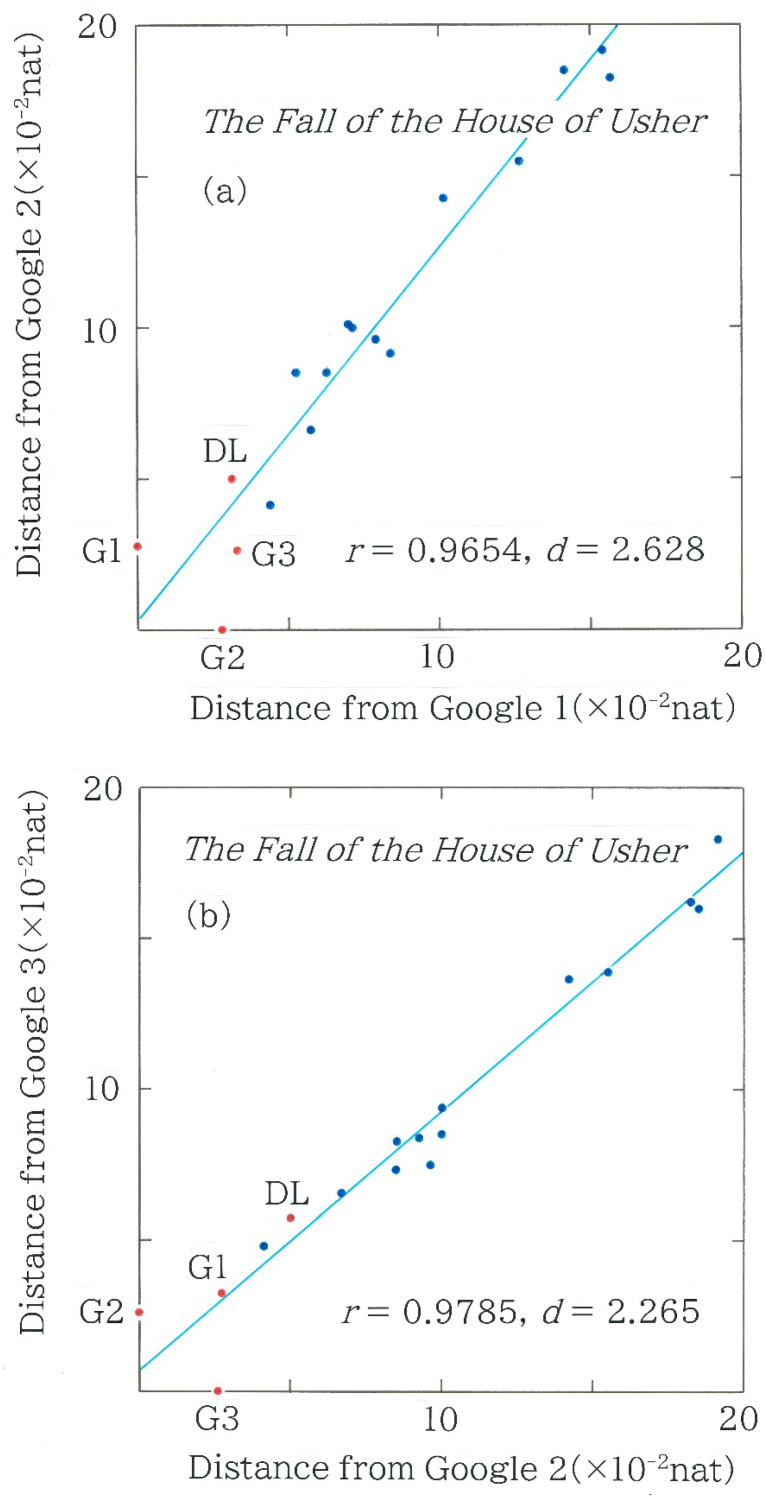

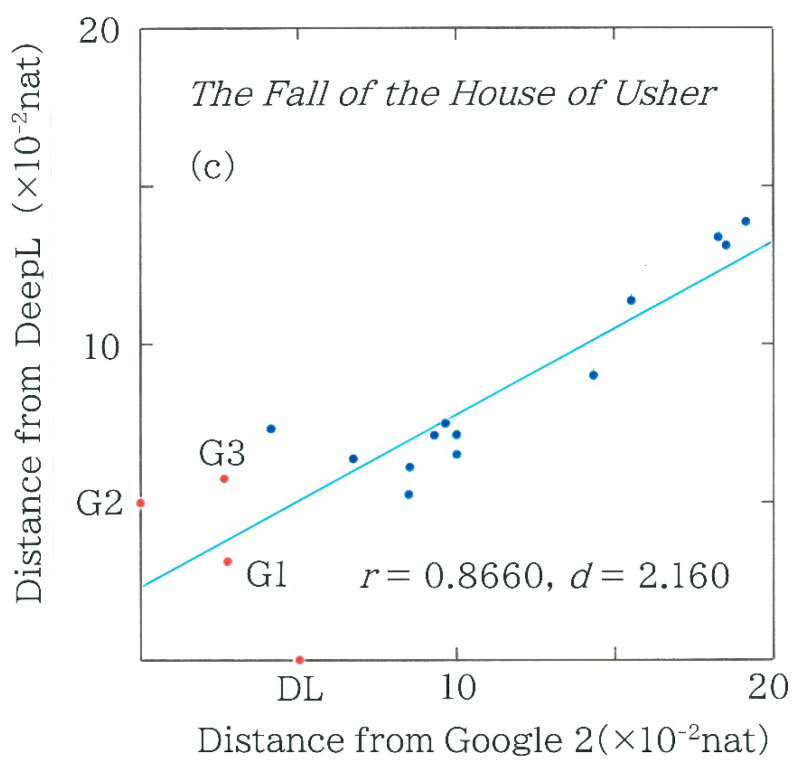
The relation between two Hellinger distances for Japanese translations of the passages from *The Fall of the House of Usher* by Edgar Allan Poe. The blue and red dots indicate the human and machine translations, respectively. The letters *r* (|*r*| ≤ 1) and *d* (0 ≤ *d* ≤ 4) denote Pearson’s correlation coefficient and the Durbin–Watson radio, respectively. The acronyms DL and G indicate DeepL (as of May 2025) and Google Translate, respectively; the number attached to G specifies the date of each machine translation: G1 (as of December 2023), G2 (as of January 2025), and G3 (as of July 2025). (**a**) Hellinger distance from G2 versus that from G1 (*y* = 1.229 *x* + 2.842 × 10^−3^). (**b**) Hellinger distance from G3 versus that from G2 (*y* = 0.8599 *x* + 5.969 × 10^−3^). (**c**) Hellinger distance from DL versus that from G2 (*y* = 0.5475 *x* + 2.288 × 10^−2^).

As the potential of the machine translation by artificial intelligence utilizing a statistically based program is steadily advancing, it is necessary to conduct a diachronic analysis for the four machine translations that were shown in Figure 7a,b. The relation between two Hellinger distances for Japanese translations of the passages from *The Fall of the House of Usher* by Edgar Allan Poe is shown in Figure 10. Here, the blue and red dots highlight the human and machine translations, respectively. The letters *r* (|*r*| ≤ 1) and *d* (0 ≤ *d* ≤ 4) denote Pearson’s correlation coefficient and the Durbin–Watson radio, respectively. The acronyms DL and G are used to indicate DeepL (as of May 2025) and Google Translate, respectively; the number attached to G indicates the date of each machine translation: G1 (as of December 2023), G2 (as of January 2025), and G3 (as of July 2025). In these plots, we notice the four features: (1) Overall there are strong correlations (0.96 < *r* < 0.98) between translations using the same device (see Figure 10a,b); (2) The strength of correlations is consistent with the period between the dates of translations; and (3) Visually, there appear three clusters, which consist of 5, 7, and 5 points; and (4) In the leftmost group there is a blue dot, corresponding to Tanizaki’s translation [10].

**Figure 11:**
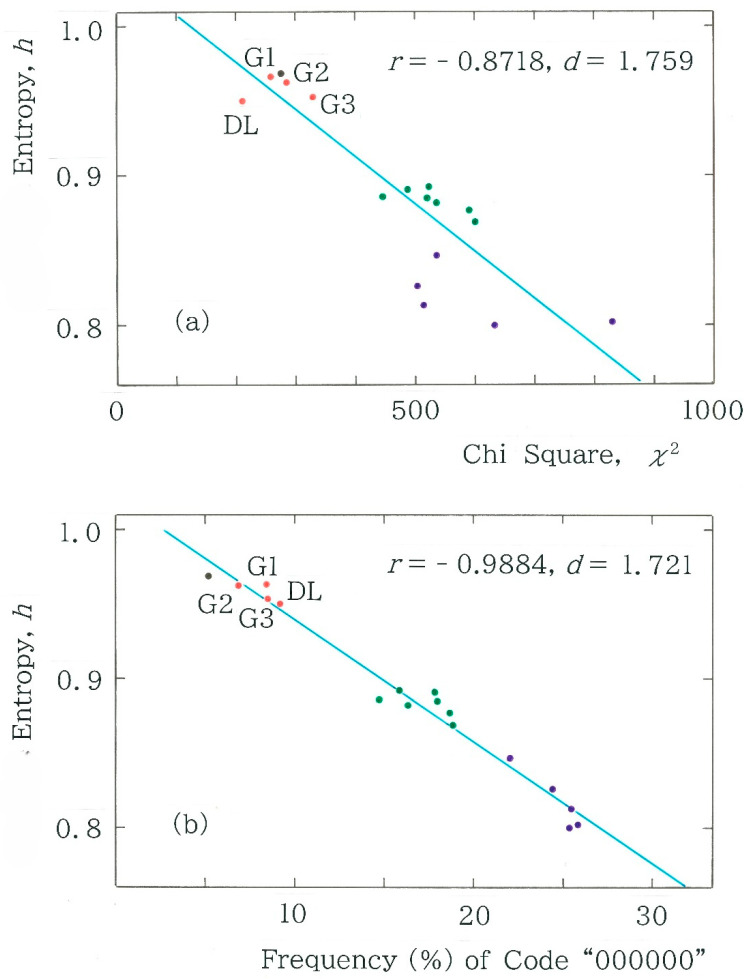
Dependence of the normalized entropy (Equation (5)) on (**a**) the chi-square value (*y* = −3.133×10^−4^ *x* + 1.035) and on (**b**) the relative frequency of the first code *C*_1_ “000000” (*y* = −8.188 × 10^−3^ *x* + 1.022). The results of the machine translations are highlighted in red. The acronyms DL and G*i* (*i* = 1, 2, and 3) denote DeepL and Google Translate, respectively. Note that there exist three clusters, which are highlighted in red, green, and purple; the single black dot in the vicinity of the first cluster corresponds to the result of Seiji Tanizaki [10].

To investigate the stability of these clusters, first we will analyze the dependence of the normalized entropy (Equation (5)) on the chi-square value (Equation (3)). The results are plotted in Figure 11a. It can be observed that although the correlation between the two characteristic values is moderate (|*r*| = 0.8718), the points (highlighted in purple) in the rightmost cluster deviate substantially from the regression line. To improve the contrast between the clusters, we will subsequently analyze the dependence of the entropy on the frequency of the dominant code *C*_1_ = 000000 instead of the chi-square value. The results are shown in Figure 11b. In comparison with those in Figure 11a, it can be seen that the regression is substantially enhanced (|*r*| = 0.9884) and, at the same time, the three clusters can be discriminated more sharply than those observed in Figure 11a. Note that the results of a cluster analysis are given in Appendix B.

### 4.3. Diversity Index Other than Entropy

In Figure 7b, for the normalized entropy, comparison was made among 17 translations, but there is a metric of diversity other than entropy. To compare the potential of the metrics, in addition to the entropy we consider the diversity index 1 − *λ* being developed by Simpson [24] in the context of ecological data analysis, where *λ* is given by

(7)  λ=∑i=164fi2n−52=∑i=164fi (fi−1)n−5n−6. 

**Figure 12:**
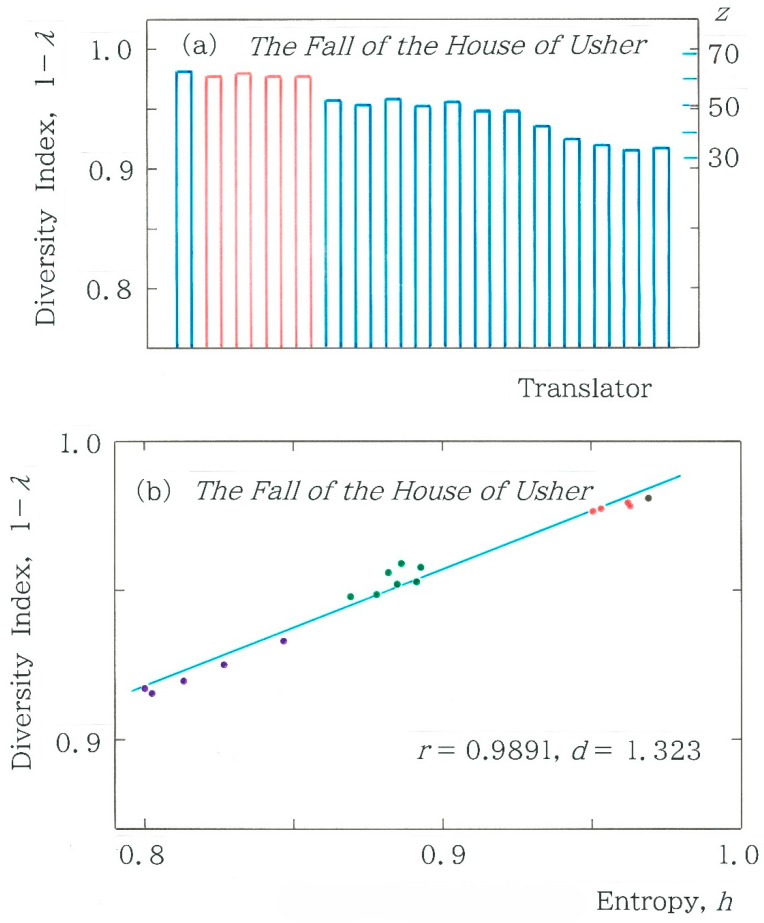
(**a**) Comparison among Simpson’s diversity indices for 17 Japanese translations of the opening paragraph in *The Fall of the House of Usher* written by Edgar Allan Poe [9]. The blue and red bars indicate the human and machine translations, respectively. In the arrangement of the 17 bars the same ordering as in Figure 7b is preserved. The numerals on the right-hand axis of ordinates indicate the standard scores defined with Equation (6). (**b**) Dependence of Simpson’s diversity index on the normalized entropy (*y* = 0.3924 *x* + 0.6041).

Here 0 ≤ 1 − *λ* ≤ 1 and the 6-bit binary coding is implied. In Figure 12a, a comparison is made among Simpson’s diversity indices for the 17 Japanese translations of the opening paragraph in *The Fall of the House of Usher*. Evidently, the discontinuity across the machine translation (in red) and the human counterpart (in blue) is reduced remarkably (Δ(1 − *λ*) = 0.0187) in comparison with the one observed in Figure 7b (Δ*h* = 0.0581). Finally, it is interesting to investigate the relationship between the two indices of diversity. Figure 12b plots the dependence of Simpson’s diversity index on the normalized information entropy. There is a strong correlation between the two indices (*r* = 0.9891) and, at the same time, there is a clear contrast emerging among the 3 clusters, but the standard deviation of Simpson’s index (*s* = 2.202 × 10^−2^) is much smaller than that of the relative entropy (*s* = 5.551 × 10^−2^). As for the cluster analysis of Figure 12b, see Appendix B.

### 4.4. Dimensionality Reduction

**Figure 13:**
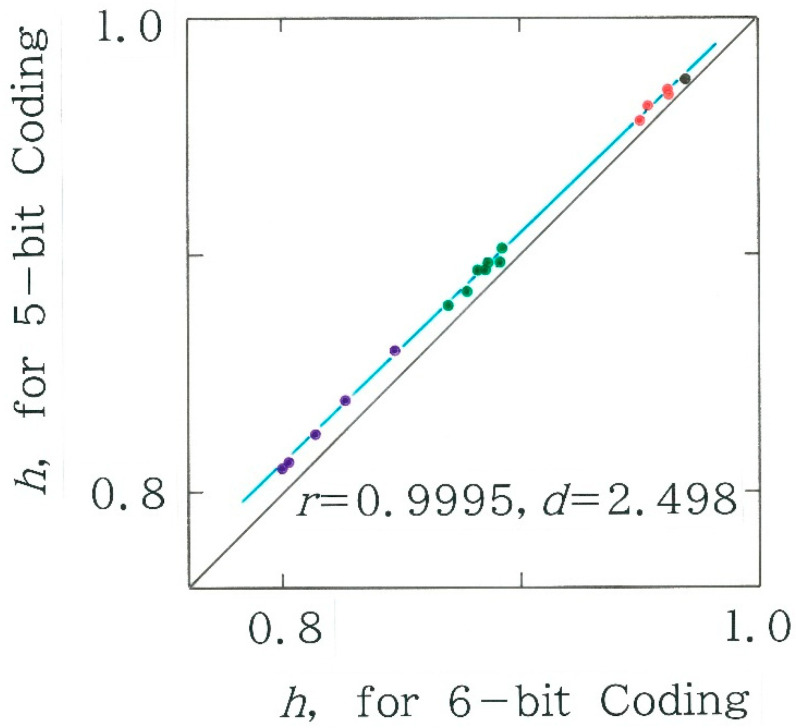
The relation between two normalized entropies for Japanese translations of the passages from *The Fall of the House of Usher* by Edgar Allan Poe (*y* = 0.9664 *x* + 0.03941). The meanings of the colors are the same as Figure 11.

Throughout this paper, we have focused on the 6-bit binary coding because this code length provides the upper limit for the lengths of our binary sequences with 480 < *n* < 1220. In other words, for our examples the chi-square testing is not applicable to the coding longer than six. In this section, computed results will be given for the 5-bit coding to investigate the robustness of our results for the 6-bit coding. First, we will revisit the results of Figure 7b, where for the 6-bit coding, comparison was made among normalized entropies for 17 Japanese translations of the opening paragraph of the Poe’s story. The results of the 5-bit coding versus the 6-bit coding are shown in Figure 13. Here, in using Equation (3) with *m* = 32, instead of Equation (4) that is valid for *m* = 64, the expected frequencies *F_i_* (*i* = 1 to 32) for *C*_1_ = 00000, *C*_2_ = 00001, …, *C*_32_ = 11111 can be calculated with the amalgamation of ‘0’ and ‘1’ in the entire binary sequence:

(8a) F1=n−4DM5 ,

(8b) Fi=n−45DM4N−M1      for i=2, 3, 5, 9, 17,

(8c) Fi=n−410DM3N−M2     for i=4, 6, 7, 10, 11, 13, 18, 19, 21, 25,

(8d) Fi=n−410DM2N−M3        for i=8, 12, 14, 15, 20, 22, 23, 26, 27, 29,

(8e) Fi=n−45DM1N−M4     for i=16, 24, 28, 30, 31,

(8f) F32=n−4DN−M5,

(8g) D=N5.

Here, *M* and *N*, respectively, are the total of ‘0’ and the grand total of ‘0’ and ‘1’. Note that for the 5-bit coding *N* = 5 (*n* − 4). Furthermore, instead of Equation (5), calculation of the normalized entropy will be carried out with the formula

(9) $$h=-\frac{\sum_{i=1}^{32}p_{i}log{\;p}_{i}}{5\;log2}.$$

**Figure 14:**
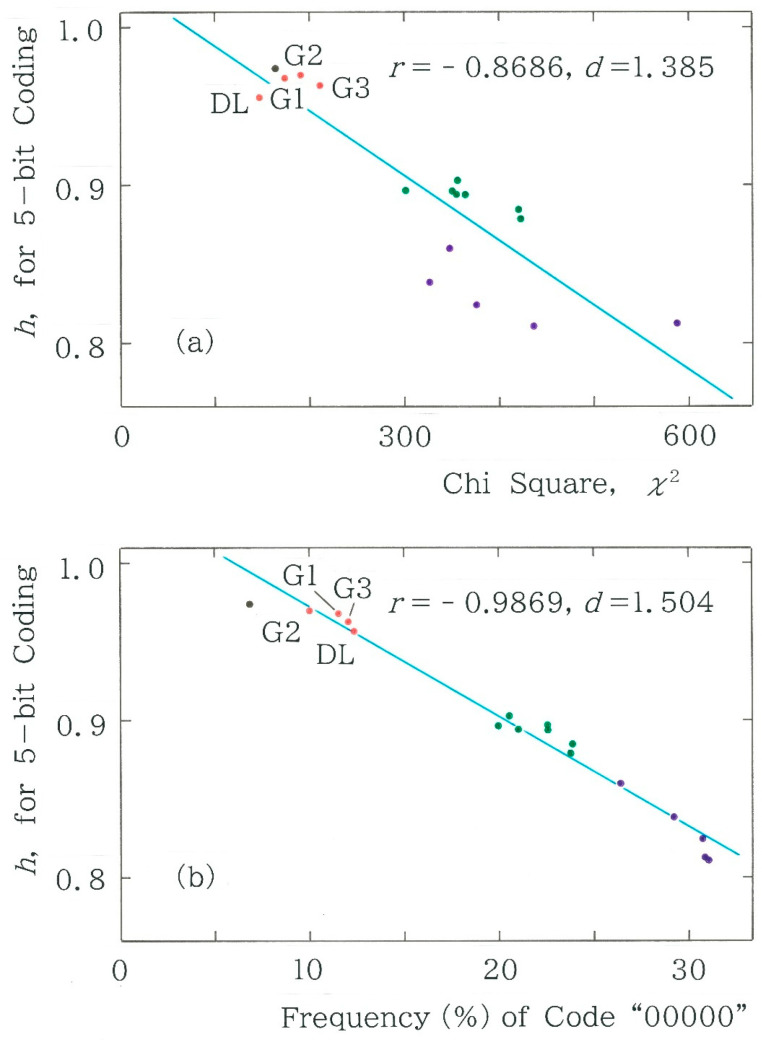
Dependence of the normalized entropy (Equation (9)) on (**a**) the chi-square value (*y* = −4.108 × 10^−4^ *x* + 1.029) and on (**b**) the relative frequency of the first code *C*_1_ “00000” (*y* = −6.993 × 10^−3^ *x* + 1.042). The results of the machine translations are highlighted in red. The meanings of the acronyms DL and G*i* (*i* = 1, 2, and 3) and the other colors are the same as Figure 11.

It can be observed in Figure 13 that there is an extremely high correlation between the two entropies (*r* = 0.9995), although for all points the value of normalized entropy for the former (on the ordinate) is slightly higher than that for the latter (on the abscissa), which can be explained by a certain averaging effect. Subsequently, the dependence of the normalized entropy on the chi-square value and the one on the relative frequency of the dominant code *C*_1_ = 00000 are shown in Figure 14a and Figure 14b, respectively. It can be seen that both plots bear a close resemblance to those observed in Figure 11, confirming the robustness in the method of coding.

## 5. Conclusions

In the context of the entropy-based time series analysis in the humanities, for several Japanese texts, using the literal-patterns-based approach to the 6-bit binary sequence we conducted a quantitative analysis of how the three characters consisting of *kanji*, *hiragana*, and *katakana* are amalgamated as an organic whole. Specifically, we chose two different texts in the former and present constitutions of Japan as well as a short story written by Edgar Allan Poe, which had been translated at least 13 times into Japanese. For the latter, a comparison was made among all human translations and the four machine translations by DeepL and Google Translate. As metrics of divergence and diversity, the Hellinger distance, chi-square statistic, normalized Shannon entropy (*h*), and Simpson’s diversity index were employed. Numerical results have shown that in terms of the entropy, the 17 translations can be divided into three clusters, and that overall, because of the entire freedom from an editorial inspection as well as a psychological bias, the machine-translated texts tend to exhibit entropy higher than the human translations that were necessarily subjected to an inspection by an editor who cannot be free from a sort of the bias. In an effort to find a compromise, texts belonging in the intermediate cluster can be regarded as possessing a familiar style of writing, where 0.85 < *h* < 0.90. The finding in this paper suggests that the present method can provide a tool useful for stylometry and author attribution. In other words, it could detect whether a certain text in question was written by a human or generated with the aid of artificial intelligence. Finally, through comparison between the two diversity indices, capabilities of the entropic measure have been confirmed. Lastly, it should be mentioned that there are limitations of our method. That is, although it can deal with a sort of readability of texts, it could not become measures for the concinnity of styles. There is further work ahead.

In addition to the two examples in the text, numerical results of the Japanese version of the periodic table of elements will be given in Appendix C.

## Figures and Tables

**Figure A1:**
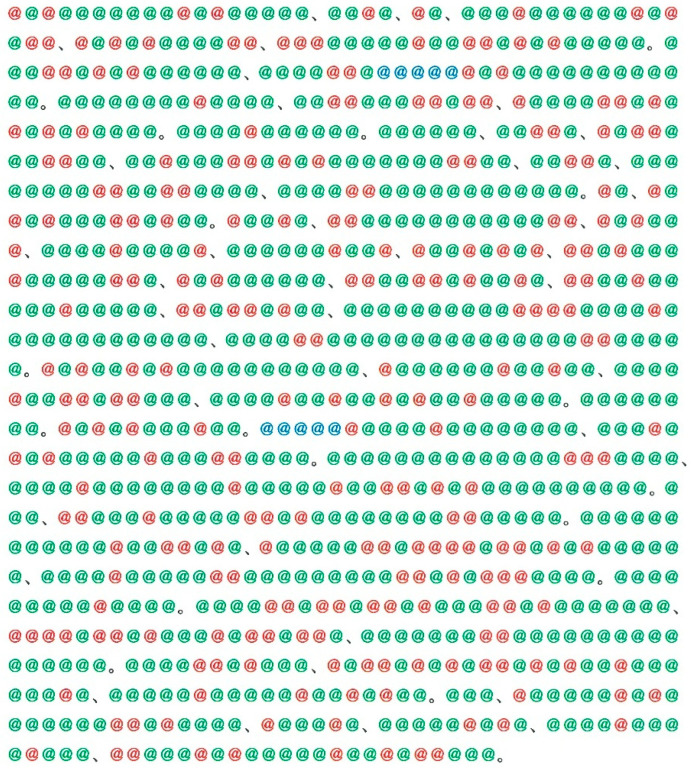
A bird’s-eye view of the renewed version of Tanizaki’s writing in 1962 [25], showing a contrast to Figure 6a (as of 1929 [10]) in the text. The meaning of tricolors is the same as the one in Figure 6.

**Figure A2:**
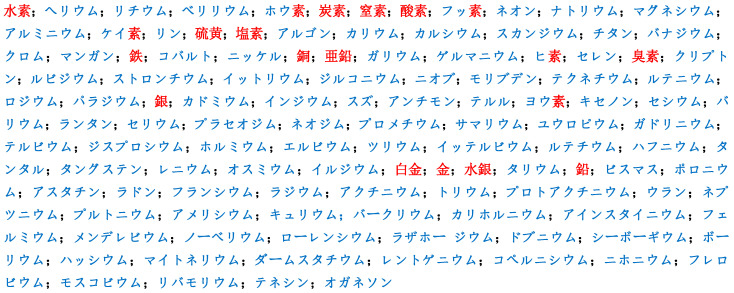
Japanese version of the periodic table of elements, which lists 118 kinds of elements (from hydrogen to oganesson) currently available. The table is written with the two forms of characters; *kanji* (Chinese characters) are marked in red, while *katakana* in blue. Note that there is no element written with *hiragana*.

## Data Availability

The raw data supporting the conclusions of this article will be made available by the author without undue reservation.

## Appendix A. Addenda to Section 3.2

After the submission of this article, the author found three additional Japanese translations of the story by Poe. Specifically, in 1962 and 2006, respectively, Tanizaki [25] and Yagi [26] had published a renewed version of their original work, while in 2022, Kawai has published a new translation [27]. Comparison of the normalized entropy shows that for Tanizaki, *h* = 0.8859 for the renewed translation, in contrast to *h* = 0.9688 for the original translation [10], while for Yagi, *h* = 0.9349, in contrast to *h* = 0.8264 for his own original [15]. It is worth noting here that through the renewal, Tanizaki’s entropy decreases substantially, whereas Yagi’s entropy increases. Indeed, it can be seen that with the renewal, Tanizaki’s point moves from Cluster 1 to Cluster 2, and at the same time, Yagi’s point moves from Cluster 3 to Cluster 1. To appeal to the eyes, the tricolor plot of the renewed text by Tanizaki [25] is given in Figure A1. Comparison of this plot with Figure 6a in the text shows a substantial increase in the rate of ‘green’ (i.e., *hiragana*), which is consistent with the trend revealed by a statistical analysis being available [23]. Lastly, Kawai’s updated translation [27] shows *h* = 0.9069, which will belong to Cluster 2.

## Appendix B. Cluster Analysis

In the visual inspection of the plot of Figure 7b, there are three groups, which allow one to define them, from the left to right, as Cluster 1, 2, and 3. Because of the steep discontinuity between the fifth and sixth bars, it is evident to identify that the size of Cluster 1 is exactly five. As for the other groups, however, their variations are relatively smooth, and consequently there is no discontinuity around the fifth bar. For this reason, we will concentrate on the analysis for Clusters 2 and 3 in Figure 11a,b as well as Figure 12b. First, with reference to the *k*-mean algorithm we write the sum of the squared Euclidean distances

$$D^{2}(k,\;12-k)=\sum_{i=6}^{5+k}[(x_{i}-{\bar{x}}_{2}{)}^{2}+(y_{i}-{\bar{y}}_{2}{)}^{2}]+\sum_{i=6+k}^{17}[(x_{i}-{\bar{x}}_{3}{)}^{2}+(y_{i}-{\bar{y}}_{3}{)}^{2}],\;\;\;\;\;\;$$

where *k* = 7 or 8; $\left({\bar{x}}_{j},\;{\bar{y}}_{j}\right)\;$ are the means of data in Cluster *j* (*j* = 2, 3); and it is assumed that the fifth point from the bottom might be an outlier [12]. Application of the formula to the three cross-sections in the text yields

$$D^{2}\left(7,\;5\right)=9.323{\times 10}^{-2},\;\;\;D^{2}\left(8,\;4\right)=8.765{\times 10}^{-2}\;\;\;\;\;\;for\;Figure\;11a,\;$$

$$D^{2}\left(7,\;5\right)=4.368{\times 10}^{-3},\;\;\;D^{2}\left(8,\;4\right)=5.702{\times 10}^{-3}\;\;\;\;\;\;for\;Figure\;11b,\;$$

$$D^{2}\left(7,\;5\right)=2.269{\times 10}^{-3},\;\;\;D^{2}\left(8,\;4\right)=2.431{\times 10}^{-3}\;\;\;\;\;\;for\;Figure\;12b.\;$$

Note that the chi-square value in Figure 11a and the relative frequency in Figure 11b are scaled as *χ*^2^/1000 and %/100, respectively. These results indicate that $D^{2}\left(7,\;5\right)>\;D^{2}\left(8,\;4\right)$ for Figure 11a, while $D^{2}\left(7,\;5\right)<\;D^{2}\left(8,\;4\right)$ for both Figure 11b and Figure 12b.

Subsequently, to confirm the validity of the number of clusters, analysis will be carried out on the assumption that there are two clusters, Cluster 1 and 2. That is, for Cluster 2

$$D^{2}\left(12\right)=\sum_{i=6}^{17}[(x_{i}-{\bar{x}}_{2}{)}^{2}+(y_{i}-{\bar{y}}_{2}{)}^{2}],$$

where $\left({\bar{x}}_{2},\;{\bar{y}}_{2}\right)\;$ are the means of data in Cluster 2. Application of the formula to the three cross-sections in the text yields

$$D^{2}\left(12\right)=1.212{\times 10}^{-1}\;\;\;\;\;for\;Figure\;11a,\;$$

$$D^{2}\left(12\right)=3.322{\times 10}^{-2}\;\;\;\;\;\;for\;Figure\;11b,\;$$

$$D^{2}\left(12\right)=1.755{\times 10}^{-2}\;\;\;\;\;\;for\;Figure\;12b.\;$$

The above results show that $Max\;\{D^{2}\left(7,\;5\right),\;D^{2}\left(8,\;4\right)\}<\;D^{2}\left(12\right)$ for the three cross-sections in Figure 11a,b and Figure 12b.

To conclude, in contrast to the validity of the (5, 7, 5) clustering of Figure 11b and Figure 12b, the plots scattered in Figure 11a should be divided into the (5, 8, 4) clusters.

## Appendix C. Japanese Version of the Periodic Table of Elements

The Japanese version of the periodic table of elements is listed in Figure A2, where *kanji* (Chinese characters) are marked in red, while *katakana* in blue. Despite the same two-tone colors being chosen, the view differs very much from that seen in Figure 3a in the text. That is, the present list exhibits a view as if red objects floated on a cobalt-blue lake. Applying our method to this sequence of characters, with the aid of Equations (2) and (5) we obtain *D_H_* = 7.119 × 10^−2^ nat and *h* = 0.3119 with *n* = 539, the latter of which is found to be substantially lower than those presented in the text (0.79 < *h* < 0.97). They say that there were several competing choices for the transcription of the periodic table [28]. For instance, suppose that *katakana* in the writing of boron (5), fluorine (9), silicon (14), phosphorus (15), arsenic (33), tin (50), and iodine (53) are replaced by *kanji*. Here the numeral in the parenthesis stands for the atomic number of each element. With this replacement, the abovementioned characteristic values become *D_H_* = 4.064 × 10^−2^ nat and *h* = 0.3450 with *n* = 534. Again, the entropy remains much lower than those observed in the text.

Incidentally, the list might be extended substantially in the future provided that unknown elements are created in an accelerator somewhere in the world. One says that theoretically, an element with the 172 atomic number will be, in principle, possible [29,30]. Even if this was achieved, the updated list will be filled much more with a parade of blue, but as long as the ‘red’ elements are preserved, entropy never vanishes. Assuming the addition of (172 − 118) × *Me* zeroes after oganesson, as un ultimate value of the normalized entropy we obtain *h* = 0.1280 (with *n* = 588) at a virtual element with its atomic number 172, where *Me* (= 5) denotes the median of the blue-word length in Figure A2.
